# Prediction Models for Successful Sperm Retrieval in Patients with Non-Obstructive Azoospermia Undergoing Microdissection Testicular Sperm Extraction: Is There Any Room for Further Studies?

**DOI:** 10.3390/jcm10235538

**Published:** 2021-11-26

**Authors:** Ettore Caroppo, Giovanni Maria Colpi

**Affiliations:** 1 Andrology Outpatients Clinic, Asl Bari, PTA “F Jaia”, Conversano, 70014 Bari, Italy; 2 Andrology Unit, Procrea Institute, 6900 Lugano, Switzerland; gmcolpi@yahoo.com

**Keywords:** non-obstructive azoospermia, sperm retrieval, male infertility, microTESE, prediction model

## Abstract

Several prediction models for successful sperm retrieval (SSR) in patients with azoospermia due to spermatogenic dysfunction (also termed non-obstructive azoospermia—NOA) have been developed and published in the past years, however their resulting prediction accuracy has never been strong enough to translate their results in the clinical practice. This notwithstanding, the number of prediction models being proposed in this field is growing. We have reviewed the available evidence and found that, although patients with complete AZFc deletion or a history of cryptorchidism may have better probability of SSR compared to those with idiopathic NOA, no clinical or laboratory marker is able to determine whether a patient with NOA should or should not undergo microdissection testicular sperm extraction (mTESE) to have his testicular sperm retrieved. Further research is warranted to confirm the utility of evaluating the expression of noncoding RNAs in the seminal plasma, to individuate patients with NOA with higher probability of SSR.

## 1. Introduction

Prediction models are widely used in the clinic to estimate the risk (or probability) of existing disease or outcome for an individual, determined by the possible values of one or more predictors. In the case of patients with azoospermia due to spermatogenetic dysfunction (also termed non-obstructive azoospermia—NOA), the probability of surgically retrieving sperm from one or both testes represents the outcome that needs to be estimated. Since the ability to predict such an outcome would allow the urologist to individuate those patients who are suited for microdissection testicular sperm extraction (mTESE), several prediction models have been developed to date, however their resulting prediction accuracy was never strong enough to translate their results to the clinical practice. Few candidate predictors have been proposed to be associated with better chances of successful sperm retrieval (SSR), but a consensus has not been reached about them. As a result, actually no clinical or laboratory factor may be used to counsel patients with NOA about their chances of mTESE success.

Indeed, there are some issues that may explain these findings. The most important one is that, in patients with NOA, the testicular parenchyma is not rarely characterized by a highly heterogeneous distribution of histologically and functionally distinct seminiferous tubules (STs), so that the retrieval of sperm is mostly dependent upon the skill and experience of the urologist, his/her learning curve being strictly correlated with the outcome of mTESE [1,2,3], rather than upon the severity of the spermatogenic dysfunction. In addition, the definition of SSR is not homogeneous among groups: ideally, SSR is defined as the retrieval of an adequate number and quality of sperm for intracytoplasmic sperm injection (ICSI); however, at least in some cases, the difference between successful (positive outcome) and failed sperm retrieval (negative outcome) may not always be as sharp as it should be to avoid the risk of misclassification. 

This notwithstanding, the number of prediction models being evaluated in this field is growing. To establish whether the current knowledge about prediction of mTESE success may justify further studies, in the present article, we will review the evidence about the predictive ability of the clinical and laboratory factors that have been previously proposed as candidate predictors of mTESE outcome.

## 2. Clinical Factors

Some prediction models of successful sperm retrieval have evaluated the predictive ability of clinical conditions that may be involved in the etiology of NOA (Klinefelter’s syndrome, Y chromosome microdeletions, cryptorchidism, varicocele), or may represent putative prognostic factors of mTESE success (testicular volume).

### 2.1. Klinefelter Syndrome

Klinefelter syndrome (KS) is the most common chromosomal abnormality in men, and is found in about 3–4% of infertile men and in more than 10% of azoospermic men [4]. KS men have typically small, atrophic testes and hypergonadotropic hypogonadism, with tubular hyalinization as the prevalent histopathological pattern. Their genetic profile is characterized in 85–90% of cases by the presence of a supernumerary X chromosome (47, XXY karyotype), while the remaining patients show a mosaic karyotype (46, XY/47, XXY), or rarely, a super-numerous sex chromosome [5]. Despite the severe spermatogenic dysfunction, 8% of patients may have sperm in the ejaculate [6], while testicular sperm may be retrieved in 20–66% of KS men by means of mTESE (see Table 1). Such a wide range of sperm retrieval rates (SRR) may be explained by the unique testicular architecture found in men with KS, who may have sperm in focal enlargements of otherwise sclerotic tubules, instead of having sperm throughout a uniformly dilated tubule [7], so that only a meticulous search within these very small testes may be successful. In addition, as summarized in Table 1, some studies suggest that SRRs may be affected by age (younger patients have better SRRs) or preoperative testosterone level (normal testosterone level is associated with better SRRs).

The predictive role of KS on SSR is still debated. A neural computational model built on 1026 men with NOA demonstrated that KS significantly predicted SSR (OR 3.07 (1.84–5.03), *p* < 0.001) [8]. On the other hand, a meta-analysis evaluating 117 studies enrolling 21,404 patients showed that SSR decreased as a function of the number of KS subjects included in the population of NOA (S = −0.02(−0.04; −0.01); *p* < 0.01) [9]. Still, different surgical methods of sperm retrievals, different surgeons and embryologists’ skill and experience, and heterogeneities in patients’ characteristics may explain such conflicting results. Further studies should clearly provide information about patients’ ages, as well as surgeon’s learning curve, to allow the correct interpretation of data.

### 2.2. Y Chromosome Microdeletions 

The global prevalence of AZF microdeletions in infertile men is estimated to be 7% (95% CL 6.74–6.79) [10]. The most frequently deleted locus in infertile men is AZFc (60–70%), followed by AZFa (0.5–4%), AZFb (1–5%) and AZFb+c (1–3%) deletion [10]. Men with complete AZFa and AZFb deletions are azoospermic, and sperm cannot be surgically retrieved [11]. A study reported that 3 out of 15 patients with AZFb deletions had sperm on mTESE [12]; however, the Authors defined the AZFb deletions using sY127 and sY134 marker, while classically, the AZFb locus is proximally defined by sY108 and distally characterized by sY134 or sY135; therefore, a partial AZFb deletion could not be excluded in such cases. Men with complete AZFc deletions may have sperm in the ejaculate or be azoospermic, but with good chances of SSR: a recent review reporting the results of 32 studies found that sperm could be retrieved in 13 to 100% of cases, particularly when mTESE was used [11]. Thus, AZFc deletion may confer better chances of SSR to patients with NOA.

### 2.3. Cryptorchidism

Cryptorchidism is considered as a reliable predictor factor of SSR in patients with NOA. A study utilizing an artificial neural network (ANN) to model the chance of SSR of 1026 men with NOA (770 training set, 256 test set) undergoing microTESE found that cryptorchidism was significant to the model [OR 2.29 (1.47–3.57), *p* < 0.0001] [8]. Sperm retrieval rates vary from 52.6% to 75% [12,13,14,15]. There is no consensus about the predictive ability of age at surgery, side (unilateral vs. bilateral) or testicular volume on SRR. Ozan and coworkers evaluated 148 patients with NOA and history of cryptorchidism undergoing mTESE, and found that SSR did not vary with age at surgery (65.1% vs. 55.4% in patients undergoing orchidopexy before or after 10 years of age respectively) or side (62.9% vs. 59.3% in patients, with unilateral of bilateral cryptorchidism, respectively) [13]. Okada et al. found that only testicular volume was predictive of SSR in a cohort of 36 formerly cryptorchid patients with NOA (OR 1.328, 95% CI 1.089–1619, *p* = 0.045) [14], while Cayan and collaborators evaluated a cohort of 327 azoospermic men with previous cryptorchidism, and found that SRR was higher in patients with total testicular volume > 13.75 mL (65.3% vs. 45.5%, *p* = 0.001), serum testosterone > 300.5 ng/dL (65.9% vs. 40.5%), serum FSH level > 17.25 mIU/mL (72.7% vs. 44.3%, *p* < 0.0001), and age at surgery < 9.5 years (70.8% vs. 42.1%, *p* < 0.0001) [15]. Well designed, multicentric studies are warranted to clarify the impact of age at surgery on the chances of SSR of formerly cryptorchid patients with NOA.

### 2.4. Varicocele

Varicocele is found in 5–10% of men with NOA [16]. Although several pathophysiological hypotheses have been proposed about the link between varicocele and NOA, no definite conclusions can be drawn [17]. Despite this, varicocele repair has been proposed to be beneficial in patients with NOA: a meta-analysis evaluating 16 studies for a total cohort of 344 azoospermic men who had undergone varicocele repair reported that 43.9% (151/344) of them had sperm in the ejaculate (sperm count was 1.82 ± 1.58 million/mL (95% CI: 0.98–2.77 millions/mL), sperm motility was 22.9% ± 15.5% (95% CI: 12.5–33.2%) 4.5 to 11 months after surgery; testicular biopsies were obtained in 8 out of 16 studies, histopathology demonstrating that the chance of having sperm in the ejaculate was significantly higher in patients with hypospermatogenesis (HS) compared to maturation arrest (MA) (OR: 2.35; 95% CI: 1.04–5.29; *p* = 0.04), and to Sertoli cell only syndrome (SCO) (OR: 12.0; 95% CI: 4.34–33.17; *p* < 0.001) [18]. However, since positive changes in the semen parameters following varicocele repair may not last forever, sperm cryopreservation is recommended [17]. The same meta-analysis reports the results of three studies evaluating the SRR in patients with varicocele, which was significantly greater in men with prior varicocele repair, compared to untreated patients (OR 2.65, 95% CI 1.69–4.14). Still, such studies were not devoid of selection bias. On the other hand, Schlegel and Kaufmann evaluated 138 patients with NOA and varicocele, 68 with a prior varicocelectomy, and 70 who did not undergo surgery: SRR was comparable in both groups (41/68 (60%) vs. 42/70 (60%), and did not vary with histopathological subcategories (26 vs. 38% in SCO, 53 vs. 47% in MA, and 96 vs. 96% in HS in patients with prior varicocelectomy or no treatment, respectively) [19]. Similarly, a study evaluating 860 patients with NOA, of whom 169 had prior history of varicocele repair, by means of a predictive model with varicocelectomy, age, prior sperm retrieval, testis volume, FSH, LH, testosterone level and diagnosis of KS as candidate predictors (all found to be predictive of SSR in univariate logistic regression), found that prior varicocelectomy was not predictive of SSR in multivariate logistic regression [20]. Given the conflicting results as above, well-designed randomized clinical trials are warranted to clarify whether varicocele repair may help in the management of patients with NOA. 

### 2.5. Testis Volume

Since seminiferous tubules contribute to approximately 80% of testis volume, this clinical parameter has been classically correlated with spermatogenesis. Indeed, a large sample size study (2.672 patients) demonstrated that testis volume correlates with sperm parameters and serum gonadotrophins levels [21], and men with testicular long axis 4.6 cm or less have been found to be more likely to have azoospermia, due to spermatogenic dysfunction [22]. Nevertheless, the correlation between testis volume and SSR in mTESE is not as intuitive as one would expect. On one hand, sperm may be retrieved even in patients with testis volume lower than 2 mL, with SRR being comparable to that of patients with larger testes (sample size = 1127 patients) [23]; on the other hand, patients with NOA due to early maturation arrest usually display normal testis volume, but have the worst chance of sperm retrieval [24]. 

Still, a meta-analysis evaluating 117 studies enrolling 21,404 patients found that testis volume significantly predicted SRR, specifically a mean volume higher than 12.5 mL predicted a SRR > 60%, with an accuracy of 86.2 ± 0.01% (*p* < 0.0001) and a specificity and sensitivity of 73% and 74% respectively; notably, the study design of the studies included in the analysis was heterogeneous with regard to patients’ clinical characteristics and the surgical procedure applied (cTESE or mTESE) [9]. Indeed, a meta-analysis that included only studies evaluating patients with NOA who had undergone mTESE (5 studies with a total of 1764 cases) found that testis volume had limited value in predicting positive sperm retrieval in patients with NOA (AUC 0.63), mostly due to low specificity (sensitivity 80%, 95% CI: 0.78–0.83, specificity 35%, 95% CI: 0.32–0.39) [25]. It may be concluded, therefore, that patients with NOA with small testes should not be discouraged from attempting mTESE in the hand of skilled urologists.

**Table 1:** Comparison of sperm retrieval rates in patients with NOA with normal karyotype or Klinefelter syndrome.

Author	Sample Size	Sperm Retrieval Rate	Predictive Factors
Ramasamy 2009 [26]	68 KS undergoing 91 mTESE	66%	Younger age associated with higher SRRs; normal T levels associated with better SRR (86%)
Bakircioglu 2011 [27]	106 KS vs. 379 nkNOA	47% in KS and 50% in nkNOA	
Sabbaghian 2014 [28]	134 KS, 537 nkNOA	28.4 in KS, 22.2% in nkNOA	T level significantly higher in patients with successful sperm retrieval
Rohayem J 2015 [29]	50 adolescent KS (13–19 years) and 85 adult KS (20–61 years)	45% in adolescent vs. 31% in adults.	LH < 17.5 and T > 7.5 nmol/L associated with the best SRR (54%)
Donker 2017 [30]	176 KS, 1423 nkNOA	28% in KS, 60% in nkNOA	
Ozer 2018 [31]	110 KS	20%	
Kizilcan 2019 [20]	81 KS, 231 nkNOA	19.7% in KS, 36.8% nkNOA (*p* = 0.006)	
Chen 2019 [32]	66 KS, 529 nkNOA	45% in KS, 44.9% in nkNOA	
Huang 2020 [33]	66 KS	36.4%	
Guo F 2020 [34]	184 KS	43.5%	Preoperative T levels affected the SRR; 134 out of 184 patients received hCG
Zhang 2021 [35]	284 KS, 485 nkNOA	44.7 in KS, 46.8% in nkNOA	
Kocamanoglu F 2021 [36]	121 KS vs. 178 nkNOA	38% vs. 55.6% (*p* = 0.012) in KS and nkNOA respectively	

hCG, human chorionic gonadotropin, nkNOA, patients with NOA with normal karyotype, KS, patients with Klinefelter syndrome, SRR, sperm retrieval rate, T, serum testosterone level.

## 3. Hormonal Parameters

Follicle-stimulating hormone (FSH) and testosterone (T) are both required to promote full spermatogenesis; in addition, their serum levels reflect both the pituitary and testicular function in physiological and pathological conditions. Indeed, the measurement of FSH and T serum levels represents the minimal initial hormonal evaluation of the azoospermic men, to distinguish between primary and secondary testicular failure [37]. Their role as predictors of spermatogenesis in patients with NOA is, however, questionable.

### 3.1. Follicle-Stimulating Hormone (FSH)

Elevated serum FSH levels are usually found in patients with non-obstructive azoospermia; however, a normal or near-normal serum FSH concentration does not always guarantee normal spermatogenesis [37]. Indeed, patients with NOA due to early maturation arrest may have low normal serum FSH level, despite having the worst chance of sperm retrieval [24]. The poor predictive ability of FSH on the chance of sperm retrieval was shown by a study on 792 men undergoing mTESE, which provided the counterintuitive demonstration that higher FSH levels were associated with greater chances of SSR [38]; a neural computational model built on 1026 men with NOA confirmed that relying on serum FSH level to counsel patients with NOA is no more accurate than flipping a coin [8]. Other studies challenged these results, but their sample size was not large enough to detect a true association. 

Since FSH level correlates with the number of spermatogonia and, to a lesser extent, primary spermatocytes [39], relying on its serum levels to counsel patients with NOA about their probability of SSR may be misleading: patients with MA and HS, as well as those with SCO with or without foci of hypospermatogenesis (focal SCO), may have comparable FSH levels, but their probability of SSR differs significantly. Indeed, a prediction model built on a development (*N* = 558) and a validation set (*N* = 695) of patients with NOA demonstrated that serum FSH level is unable to predict histopathological subcategories such as MA and focal SCO, and has low sensitivity (40.9%) and specificity (46.8%) in predicting HS and SCO, respectively [40]. These data reinforce older data against the use of basal FSH level as a predictor of SSR in patients with NOA, and should discourage further evaluation of serum FSH as marker of residual spermatogenesis in these patients.

### 3.2. Testosterone

Testosterone (T) signaling is required for spermatogenesis to proceed beyond meiosis. Consequently, it has been postulated that patients with hypogonadism (serum T < 300 ng/dL) may have lower chances of SSR compared to patients with normal serum T levels. Indeed, a pooled estimate of six studies evaluating 2029 patients with NOA undergoing mTESE demonstrated that patients with normal T levels had a significantly higher chance of SSR compared to those with subnormal T levels (OR 1.63, 95% CI 1.08–2.45, *p* = 0.02) [41]. However, the available evidence has provided conflicting results. Reifsnyder et al. evaluated 736 men undergoing mTESE; 348 (47.3%) with baseline T level < 300 ng/dL and 388 (53%) with baseline testosterone levels greater than 300 ng/dL. Among patients with hypogonadism, 88% received hormonal treatment. SRR did not vary among men with low vs. normal baseline T levels; yet, the mean presurgical T level was normal in patients with previous low baseline T levels as the effect of hormonal treatment. Moreover, 18% of patients receiving hormonal treatment did not respond to treatment, but their SRR was comparable to that of responders to treatment [42]. Enatsu et al. evaluated 329 patients, of whom 65 had KS, and found that serum T levels did not differ among men with SSR (97) and SRF (232) (420 + 180 vs. 430 + 190 ng/dL; *p* = 0.42) [43]. Althakafi et al. evaluated 421 patients, of whom 181 had low baseline T levels, and found no difference in SRR between those with normal and low T levels (SRR 38.6% vs. 40.3%, *p* = 0.718). Fifty patients received hormonal treatment with clomiphene citrate (CC) or human chorionic gonadotropin (hCG) due to subnormal T levels: their SRR was comparable to that of patients with normal baseline T levels (36% vs. 38%, *p* = 0.736) [44]. Kizilkan et al. evaluated 860 patients and found that T levels were predictive of SSR in univariate, but not in multivariate, logistic regression [20]. On the other hand, Mehmood et al. and Çayan et al., evaluating 264 and 327 patients respectively, found that SRR was significantly lower in men with low baseline T levels compared to those with normal baseline T levels (40.6 vs. 57.25, *p* = 0.0068, and 40.5% vs. 65.9%, *p* < 0.0001 respectively) [15,45]. Accumulating evidence suggests that higher baseline T levels may be associated with a higher probability of SSR in men with KS [26,34]. 

It has been suggested that intratesticular testosterone (ITT) measurement could represent a more reliable way of assessing the role of testosterone on the probability of SSR in men with NOA. Due to the inherent risks of performing testicular aspiration to obtain a direct assessment of ITT level, a measurement of the circulating levels of 17-hydroxyprogesterone (17OHP) has been proposed as an indirect biomarker of ITT levels, since 17 OHP is likely to be of testicular and not adrenal origin in men. Indeed, serum 17 OHP levels were found to be undetectable in men receiving exogenous testosterone replacement therapy, and to increase after CC and hCG treatment [46]. Studies evaluating the predictive ability of serum 17 OHP on the probability of SSR in patients with NOA are needed to provide evidence in support or against such a hypothesis.

## 4. Testis Histology

There is great consensus about the close relationship between different histopathological categories and mTESE outcome: patients with SCO have the lowest probability of SSR (22.5–41%), while patients with HS have the best chances of sperm retrieval (73–100%), and patients with late MA have better prognosis (SRR 27–86%) compared to those with early MA (SRR 27–40%) [47]. Indeed, a meta-analysis evaluating 19 articles showed that HS predicted SSR (pooled diagnostic odds ratio (DOR) 16.49, 95% CI: 9.63–28.23) with a sensitivity of 30% and specificity of 98%, AUC 0.6758; SCO had a negative predictive ability on SSR (AUC 0.27), while MA had a poor predictive accuracy (AUC 0.55) [25]. 

To obtain a realistic picture of the severity of spermatogenic dysfunction, the testicular specimen sent to the pathologist should be representative of the overall appearance of the testicular parenchyma. However, it is not uncommon for men with NOA to have more than one histopathological report. Very recently, Punjani et al. demonstrated that these patients may display up to four distinct histopathological subcategories, the increasing histopathological variety being associated with a higher probability of SSR (SRR was 33% in men with one histopathological subtype, compared to 94% in men with 4 subtypes) [48].

Testis histology has been found to be predictive of SRR also in men undergoing salvage mTESE after a failed surgical attempt. Despite previous surgery possibly harming the blood supply of the testis with a potential risk of testicular tissue damage, Tsujimura [49] and Kalsi et al. [50] found comparable SRRs in patients undergoing salvage mTESE after failed cTESE stratified according to testis histology (39% and 40% in SCO, 41.7% and 36% in MA, and 100% and 75% in HS, respectively). Data from Xu et al. [51] confirmed that HS associates with high SRR (85%) even in patients with previous sperm retrieval attempts, but found lower SRR in patients with SCO (5.5%) and MA (25%) compared to previous reports. Very recently, our group found that early and late MA were associated with the lowest probability of SSR (8.7 and 11.1%, respectively), while sperm was retrieved in 85% of men with HS; SRRs in patients with SCO differed significantly according to the presence (focal SCO) or not (complete SCO) of residual areas of HS (SRR 100% vs. 24.4%, respectively) [52].

The obvious limit of testis histology is that it may be obtained only after surgery, therefore it may be used to counsel patients about the probability of having their testicular sperm retrieved in further surgical attempts. In occasional situations, however, testicular histology may be available when a diagnostic testicular biopsy has been done prior to microTESE, and there may be of help in the counselling of patients with NOA.

## 5. Molecular Markers Expression in the Seminal Plasma

Given the limited accuracy of hormonal and clinical parameters in predicting the probability of SSR in patients with NOA prior to surgery, researchers have sought to evaluate the feasibility of using the expression of some molecular markers in the seminal plasma as markers of residual spermatogenesis in such patients. 

The evaluation of germ cell-specific mRNAs as predictors of SSR in patients with NOA has brought conflicting results. Following the demonstration that the testicular expression of ESX1, an X-linked homeobox gene, was restricted to germ cells, particularly the spermatogonia/preleptotene spermatocytes and round spermatids, and correlated with SSR [53], a group of researchers found that the seminal plasma levels of ESXI were significantly lower in men with NOA compared to normozoospermic subjects (*p* < 0.0001), and predicted SSR in men with NOA with a sensitivity of 84%, but with a specificity of 28% [54]. However, in a further study, the seminal plasma of ESXI was found to be comparable among men with NOA and normozoospermic men [55]; on the other hand, the seminal plasma levels of protamine-1 (PRM1) were found to predict SSR with a sensitivity of 89%, and a specificity of 90%. In another study, however, seminal plasma of PRM1, together with PRM2, DAZ and AKAP4, although being undetectable in patients with SCO, could not predict SSR [56]. Finally, several studies have evaluated the predictive ability of seminal DDX4 mRNA expression on SSR, but again, with conflicting results [reviewed in [57].

Seminal plasma also contains high concentrations of extracellular vesicles that are consistent with exosomes, which originate from the male reproductive tract, and contain coding and noncoding RNAs that vary according to their origin, enabling them to (hypothetically) reflect the pathophysiological conditions of the organ of origin. Some microRNAs (miRNAs) have been found to be preferentially expressed and localized to spermatocytes and spermatids (miR-34b/c and miR-449) or late-stage male germ cells (miR-122), and to be differentially expressed in testis biopsies of patients with and without elongated spermatids (miR-449a, miR-34c-5p and miR-122) [58]. A study evaluated the expression of exosomal miRNAs in the seminal plasma of infertile men with NOA or obstructive azoospermia, demonstrating that three miRNAs, miR-31-5p, miR-539-5p and miR-941, were downregulated in patients with obstructive azoospermia compared to men with NOA. The further evaluation of 12 patients with NOA with (*N* = 8) or without (*N* = 4) SSR showed that the association of the expression values of miR-539-5p and the miR-941 was predictive of SSR [59]. However, due to the very small sample size of such a study, further studies are warranted to provide conclusive results. Indeed, another study found that miR-539-5p was not predictive of SSR, nor could it discriminate normozoospermic, oligozoospermic, and azoospermic men from each other [60].

Long noncoding RNAs (lncRNAs) have been found to play a critical role in spermatogenesis: specifically, they have been implicated in regulating protein-coding genes at the epigenetic level, and it has been speculated that these germ-specific lncRNAs may be involved in epigenetic regulation during spermatogenesis [61]. Many of them display restricted expression in the testis, thus enabling their use as noninvasive biomarkers of spermatogenesis in men with NOA. A recent study investigated the predictive ability of extracellular vesicle long noncoding RNAs (exlncRNAs) in patients with NOA: after having selected 16 exlncRNAs on the basis of their different expression in normozoospermic and azoospermic patients, the Authors evaluated their diagnostic accuracy in predicting SSR in 30 patients with NOA who had (*N* = 18) or not (*N* = 12) their testicular sperm retrieved by mTESE. The Authors built a prediction model based on 9 exlncRNAs (LOC100505685, SPATA42, CCDC37-DT, GABRG3-AS1, LOC440934, LOC101929088, LOC101929088, LINC00343 and LINC00301) and found that it predicted the probability of SSR with a sensitivity of 88.9% and a specificity of 100%, AUC 0.986. The model was then validated on 66 patients with NOA, with a resulting AUC of 0.960 [60]. Further studies are, however, warranted to validate the findings of the present study, and to confirm or challenge the predictive ability of other molecular markers expressed in the seminal plasma.

## 6. Conclusions 

The available evidence suggests that no patient with NOA should be discouraged from attempting mTESE, based on the clinical and laboratory parameters that have been tested to date as candidate predictors of SSR. Azoospermic men with complete AZFc deletions and history of cryptorchidism may have better chances of SSR compared to those with idiopathic NOA, while the predictive role of KS on SSR is still debated. While serum FSH level and testis volume are hardly informative about the presence of residual foci of spermatogenesis in patients with NOA, it could be interesting to assess the predictive role of markers of intratesticular testosterone level (such as serum 17 OHP) on SSR. Future studies are also required to evaluate the feasibility of molecular markers in the seminal plasma, particularly non-coding RNAs, as markers of residual spermatogenesis in patients with NOA.

## Data Availability

Not applicable.
